# Supplementary material for: Splam: a deep-learning-based splice site predictor that improves spliced alignments
Source: Genome Biol. 2024 Sep 16;25:243. doi: 10.1186/s13059-024-03379-4 (PMC11406845; doi:10.1186/s13059-024-03379-4)
Supplement: Supplementary file 1 — Additional file 1: Supplementary Note S1-S4, supplementary Fig S1-S15, and supplementary Table S1-S12. [file 13059_2024_3379_MOESM1_ESM.docx]

**Supplementary Notes**

**Note S1.** Results of benchmarking Splam to other splice site predictors

We benchmarked Splam against SpliceAI, Pangolin, and Spliceator. To evaluate all tools, we compiled a test set consisting of 1,000 splice junctions each from the Positive-MANE and Positive-Alt datasets, along with 2,000 junctions from the Negative-1 dataset, totaling 4,000 splice junctions. This dataset included junctions only from chromosomes 1 and 9, which were excluded from training for all tools.

We ran SpliceAI with the same settings as described in the main paper. SpliceAI's output includes probabilities that indicate whether a position in the input sequence is a donor, acceptor, or non-splice site. We determined the final donor and acceptor scores for each splice junction by computing the average probabilities that were obtained from five SpliceAI model runs. Pangolin produces four predictions for four different specific tissue types; therefore we average these four predictions in order to compare Pangolin to the other systems. We ran Spliceator with a window size of 400 nt in order to match Splam’s input size.

We compared the performance of Splam, SpliceAI, Pangolin, and Spliceator using precision-recall curves (Fig S1). For both the donor and acceptor site predictions, Splam achieved an AUC of 0.99 and, while SpliceAI achieved an AUC of 0.98. Spliceator achieved an AUC of 0.85 for the donor site and an AUC of 0.59 for the acceptor site, while Pangolin achieved AUCs of 0.77 and 0.82 for the donor and acceptor sites, respectively. Based on these results, we focused on comparisons with SpliceAI for the remainder of our analysis.

**Note S2.** Evaluation of Splam on unannotated splice junctions extracted from GTEx RNA-Seq samples with varying levels of spliced alignment support.

We collected unannotated splice junctions from 9,795 GTEx RNA-Seq experiments used to create the CHESS human database^1^, supported by varying numbers of spliced alignments, ranging from 2 to 100 reads. We chose junctions from chromosome 1 and chromosome 9, which were excluded from the training dataset. The datasets are named Novel-2, Novel-3, etc. up to Novel-100, indicating the number of supporting spliced alignments. This selection process was intended to assess Splam's performance across a wide array of negative splice junctions, from those highly unlikely to be true splice sites (with just 2-3 reads supporting them) to those with much greater support.

We applied Splam to score each dataset, from Novel-2, Novel-3, and all subsequent datasets through Novel-100. The donor and acceptor score distributions for selected datasets (Novel-2, Novel-20, Novel-40, Novel-60, Novel-80, and Novel-100) are presented in Fig S11. As the number of spliced alignments supporting the splice junctions increases, there is a more notable peak at the score of 1.0, indicating that Splam is more likely to identify these sites as genuine splice donors or acceptors.

Next, to compare splice sites with high Splam scores to those with low scores, we set a threshold of 0.5 and quantified the proportion of splice junctions that Splam predicted as positive. Fig S12a shows that Splam predicts a greater number of splice junctions as positive when they are supported by a higher number of spliced alignments. We also calculated the average donor and acceptor site scores for each Novel-x group and plotted them against the number of spliced alignments (Fig S12b). The mean splice site scores are also proportional to the number of spliced alignments. These result show that as the number of spliced alignments increases, Splam is more likely to assign a high score to the junction, despite Splam being trained solely on DNA sequences without any coverage information.

**Note S3.** Training Splam across various input sequence lengths

In this study, we introduced a novel approach where the system learns to recognize paired donor and acceptor sites, using windows of 400 nt centered on each site. We concatenated these pairs to form 800 nt input sequences for training Splam.

In this experiment, our goal was to understand how the input window size influences performance. Consequently, we varied the input sequence lengths to 40, 100, 200, 400, 600, and 800 nucleotides, and trained a separate Splam model on each. We designated the models trained with different sequence lengths as Splam-40, Splam-100, Splam-200, Splam-400, Splam-600, and Splam-800, respectively.

For the model training, we randomly selected 20,000 splice junctions from each of the Positive-MANE and Positive-Alt, as well as 40,000 splice junctions from each of the Negative-Random and Negative-1. The training dataset includes splice junctions from all chromosomes, except Chromosome 1 and 9. Similarly, for testing the model, we randomly selected 20,000 splice junctions from each of the Positive-MANE and Positive-Alt, along with 40,000 splice junctions from each of the Negative-Random and Negative-1. The testing dataset exclusively contains splice sites located on Chromosome 1 and 9. We used the Splam model architecture with five residual groups (Figure 1).

Each Splam model was trained for 15 epochs with a batch size of 100. The testing dataset, comprising 60,000 splice sites, was evaluated after each epoch, with each epoch consisting of 600 steps, resulting in a total of 9,000 steps. At each step, we calculated the AUPRC (Area Under the Precision-Recall Curve), precision, and recall metrics for both donor (Fig S13a) and acceptor sites (Fig S13b). We then applied a moving average with a window size of 40 to each metric, utilizing NumPy's convolution function for smoothing. This approach helps to observe the trend more clearly.

We observed that the model took longer to recognize splice sites with increased input sequence length, requiring additional update steps to process the greater volume of information and learn the correct patterns. After 15 epochs of training, the performances of the Splam-200, Splam-400, Splam-600, and Splam-800 models converged to similar levels (Fig S13). In contrast, the Splam-100 and Splam-40 models exhibited slightly lower top-k accuracy, AUPRC, and precision. This suggests that at least a 200-nucleotide flanking sequence is needed for accurate performance. We selected Splam-800 as the final model because it encompasses the most information, but further research is needed to determine whether a smaller window size might be superior.

**Note S4.** Ablation study of the Splam model architecture

To evaluate the robustness of the Splam model architecture, we conducted an ablation study by sequentially removing residual groups to observe the impact on performance across training and testing datasets. The Splam model comprises five residual groups, with each group containing four residual units. In our experiments, 'RSG_X' designates a configuration with X residual groups. Thus, the original model is designated 'RSG_5'.

For training the models, we randomly selected 20,000 splice junctions each from the Positive-MANE and Positive-Alt datasets, and 40,000 splice junctions each from the Negative-Random and Negative-1 datasets. The training dataset includes splice junctions from all chromosomes, except Chromosome 1 and 9. Similarly, for testing we randomly selected 20,000 splice junctions each from Positive-MANE and Positive-Alt, and 40,000 splice junctions each from Negative-Random and Negative-1. The testing dataset exclusively contains splice sites located on Chromosome 1 and 9.

In our experiments, we varied the number of residual groups between one and five. Each Splam model was trained for 15 epochs with a batch size of 100. The testing dataset, comprising 60,000 splice sites, was evaluated after each epoch, with each epoch consisting of 600 steps, resulting in a total of 9,000 steps. At each step, we calculated the AUPRC (Area Under the Precision-Recall Curve), precision, and recall metrics for both donor (Fig S14a) and acceptor sites (Fig S14b). We then applied a moving average with a window size of 40 to each metric, utilizing NumPy's convolution function for smoothing. This approach helps to observe the trend more clearly.

We observed that as the number of residual groups increased, the model's performance improved, as expected. The performance of RSG_4 (represented by the red curve) and RSG_5 (represented by the purple curve) was similar, suggesting that at least four residual groups are required for optimal accuracy. We chose the 5-residual group model architecture as the final model.

**Supplementary Figures**

**Fig S1:** Precision-recall curves comparing Splam (blue curve), SpliceAI (orange curve), Spliceator (green curve), and Pangolin (red curve) for donor (a) and acceptor (b) splice site predictions. The analysis was performed on a test dataset comprising 2,000 positive and 2,000 negative splice junctions.

**Fig S2: (a)** The inputs and outputs for Splam, SpliceAI, and SpliceAI-10k-Ns for each splice junction. At the top is the input for Splam, with 400nt flanking the donor site and another 400nt flanking the acceptor site. The output is a set of labels for the 800nt, shown in yellow. The second row represents the input to SpliceAI in its standard configuration, with 200nt upstream and downstream of the donor and acceptor sites, the entire intron regardless of its length, and 10Kb of flanking sequence. The 200nt upstream and downstream sequences prevent the donor and acceptor sites from being the boundary nucleotides. The output of SpliceAI is similarly a set of labels for the region shown in yellow. The third row represents a slightly modified input designed for this study where the 5Kb flanking sequences are replaced with Ns, which we call SpliceAI-10k-Ns. **(b)** The amount of sequence used as input by Splam, SpliceAI, and SpliceAI-10k-Ns for the Positive-MANE and Positive-Alt datasets. Blue regions represent intronic sequences and orange regions represent flanking/exonic sequences. The ratio between the input size for Splam and SpliceAI is shown at the top of each bar. For Positive-MANE, the ratios of SpliceAI and SpliceAI-10k-Ns to Splam are 14.0 and 7.7, respectively. For Positive-Alt, the ratios of SpliceAI and SpliceAI-10k-Ns to Splam are 20.7, respectively.

**Fig S3:** The splice junction score distribution for Splam, SpliceAI, and SpliceAI-10K-Ns. 10,000 splice junctions were randomly selected from the Positive-MANE, Positive-Alt, Negative-1, and Negative-Random test datasets, totaling 40,000 splice junctions. In the plot, each dot represents a splice junction, where the x-axis corresponds to the donor score and the y-axis represents the acceptor score. The dots are color-coded based on clustering density, with yellow indicating the highest density and blue indicating the lowest density.

**Fig S4: (a)** Kernel density plots visualizing the differences between donor and acceptor scores (donor score − acceptor score). In **(b)** the score differences between -0.15 and 0.15 have been removed to provide a closer look at the splice junctions where the scores of donor and acceptor sites were relatively large. The green density plot represents Splam and the blue density plot represents SpliceAI-10k-Ns.


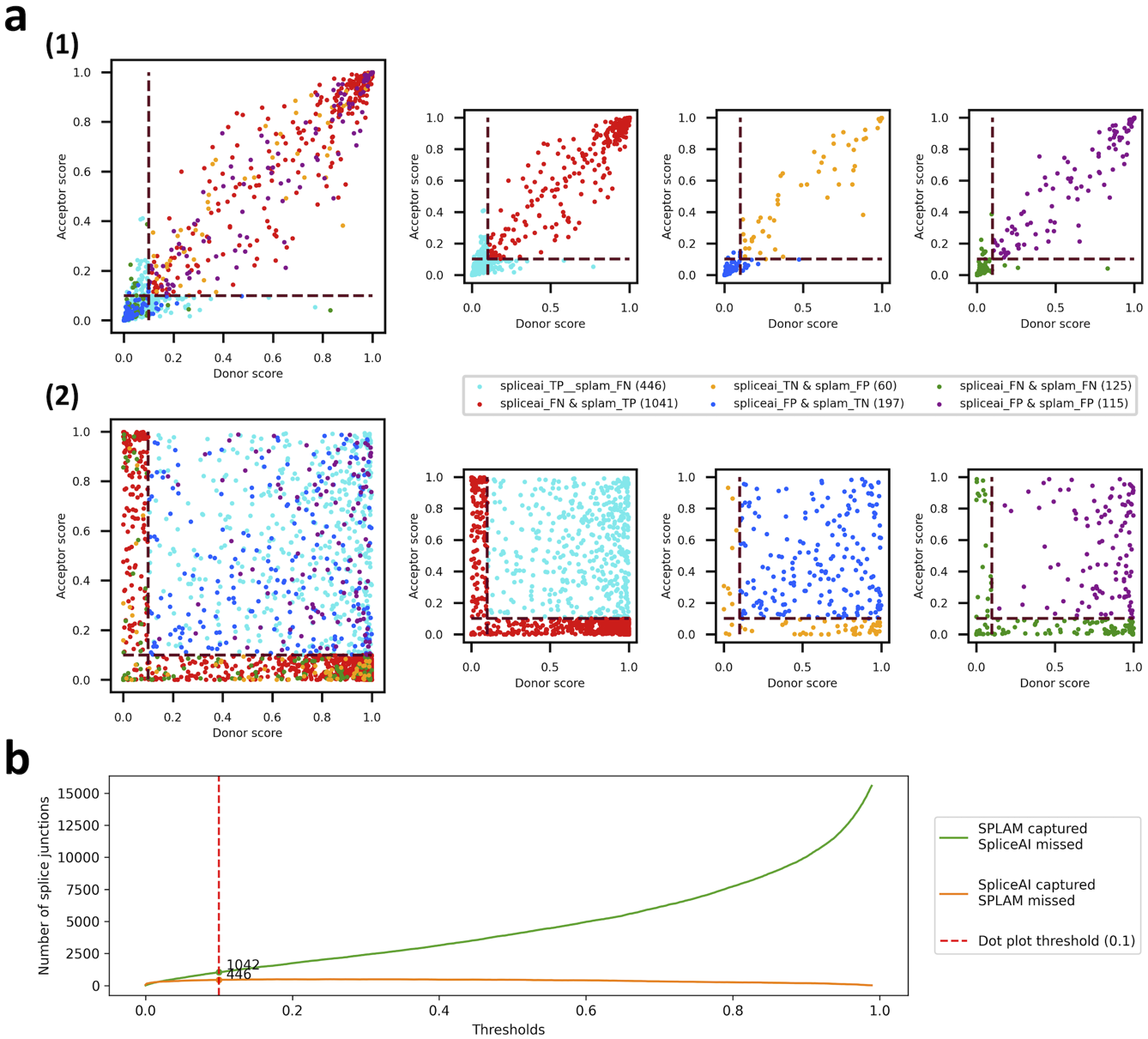


**Fig S5:** The results of comparing Splam and SpliceAI-10k-Ns. In Figure **(a)**, scatter plots **(1)** and **(2)** are depicted for Splam and SpliceAI-10k-Ns, where each dot represents a junction. The x-axis represents the donor score, while the y-axis represents the acceptor score. The red dashed lines indicate the cutoff threshold lines that determine whether a donor or acceptor is classified as good or bad. True positives (TPs), true negatives (TNs), false positives (FPs), and false negatives (FNs) are plotted based on the scores assigned by Splam **(1)** and SpliceAI **(2)**. The red dotted lines illustrate the thresholds used to label a point as true or false. To maintain clarity, these plots exclude the numerous points where both programs made correct predictions. The smaller subplots in the second and third columns display subsets of junctions where one program was correct while the other was incorrect (TP and FN, or FP and TN). The fourth column presents subsets of junctions where both programs made incorrect predictions (FP and FN). **(b)** The number of splice sites where Splam and SpliceAI disagree is shown. The green curve represents the instances where Splam correctly predicts them as true positives, but SpliceAI predicts them as false negatives. The orange curve represents the cases where SpliceAI correctly predicts them as true positives, but Splam predicts them as false negatives. The x-axis displays the threshold range from 0.001 to 0.999, while the y-axis represents the number of splice junctions. The vertical dashed red line signifies the 0.1 threshold, which is highlighted in **(a)**.

**Fig S6:** The number of splice junctions where Splam and SpliceAI disagree with each other or both programs get the wrong predictions. **(a)** The results comparing Splam and SpliceAI-10k. **(b)** The results comparing Splam and SpliceAI-10k-Ns. The blue curve represents the instances where Splam correctly predicts them as true positives, but SpliceAI predicts them as false negatives. The orange curve represents the cases where Splam correctly predicts them as true negatives, but SpliceAI predicts them as false positives. The green curve represents the cases where SpliceAI correctly predicts them as true positives, but Splam predicts them as false negatives. The red curve represents the cases where SpliceAI correctly predicts them as true negatives, but Splam predicts them as false positives. The purple curve represents both Splam and SpliceAI wrongly predicting them as false negatives, and the brown curve represents both Splam and SpliceAI wrongly predicting them as false positives. The x-axis displays the threshold range from 0.001 to 0.999, while the y-axis represents the number of splice junctions.


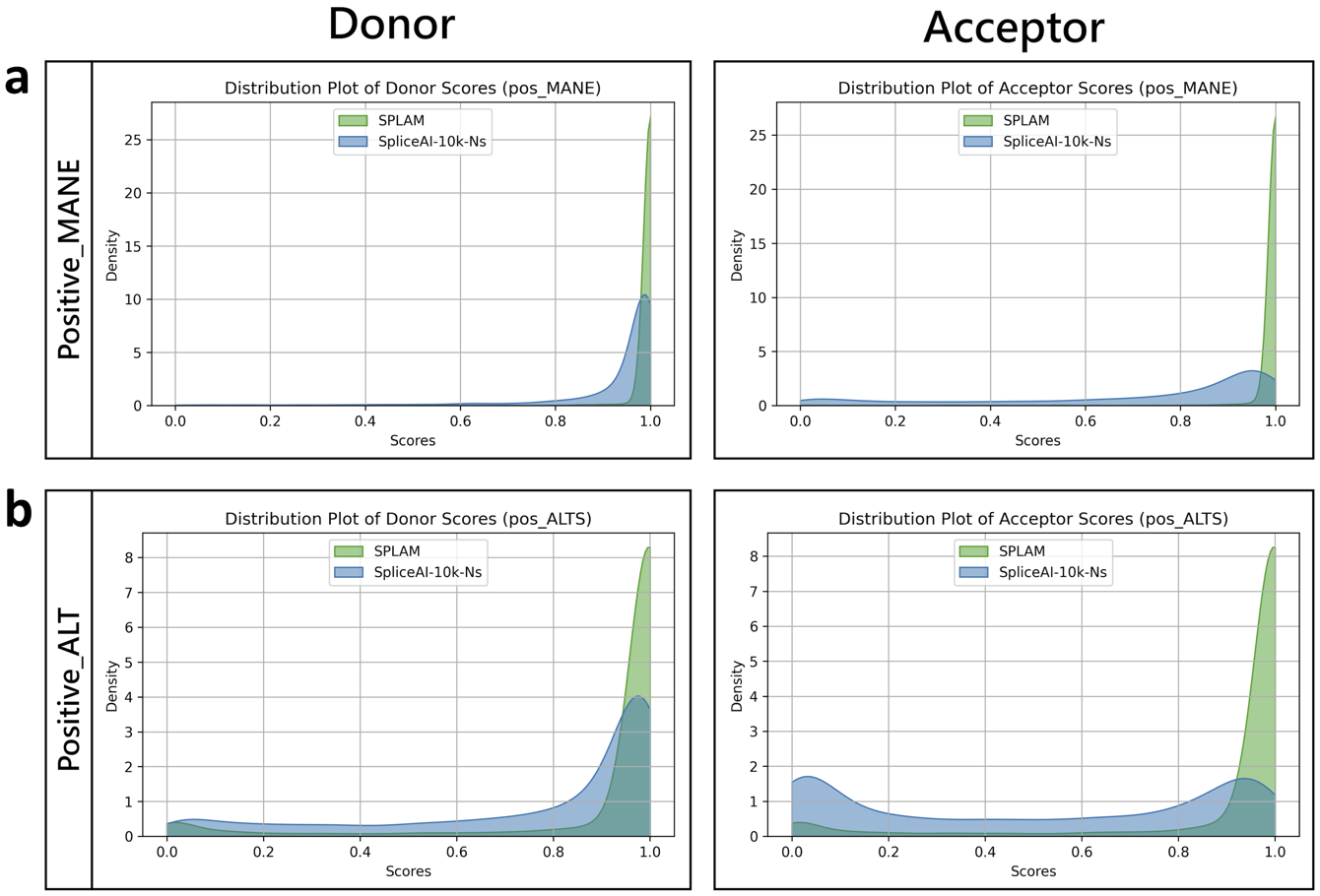


**Fig S7:** The score kernel density plots for Splam and SpliceAI-10k-Ns. The x-axis is the scores ranging from 0.0 to 1.0. The green kernel density plot represents Splam, and the blue kernel density plot represents SpliceAI-10k. **(a)** The first row shows the results of Positive-MANE, and **(b)** the second row shows the results of Positive-Alt. The first column represents the score distribution of donor sites from all splice junctions in the testing dataset, whereas the second column represents the score distribution of the acceptor sites from all splice junctions.


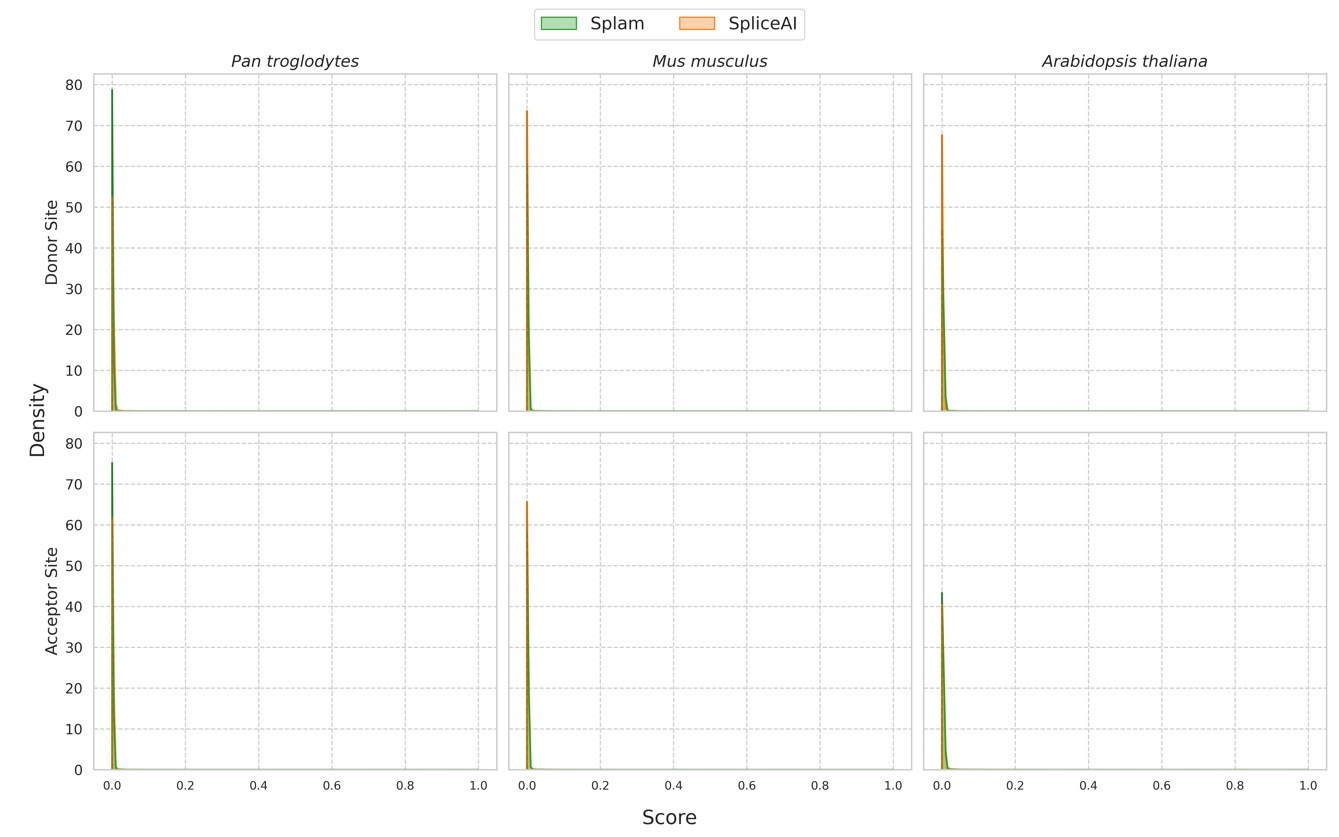


**Fig S8:** Comparison of score distributions for Splam and SpliceAI when applied to 25,000 randomly chosen splice sites from chimpanzee (left), mouse (center), and *Arabidopsis* (right). Results for donor sites are shown across the top, and acceptor sites on the bottom. Scores assigned by each program are plotted along the x-axis, while densities for both donor and acceptor sites are plotted on the y-axis. The scores for both Splam (green) and SpliceAI (orange) exhibit a narrow distribution that peaks near 0.0, indicating that the vast majority of random GT-AG splice-junction-like sequences on the reverse strand of protein-coding genes are assigned low scores, with *Arabidopsis thaliana* having a relatively lower peak.

**Fig S9:** The ROC and PR curves of Splam, SpliceAI, and SpliceAI-10k-Ns were evaluated on test sets of splice junctions from three different species: chimpanzee (*Pan troglodytes*), house mouse (*Mus musculus*), and flowering plant *Arabidopsis thaliana*. Each test set consisted of 25,000 randomly selected splice junctions from its annotation file and 25,000 randomly generated pseudo-splice junctions. The pseudo-splice junctions were created by randomly selecting GT-AG pairs on the reverse strand of protein-coding genes (see Methods). The first row displays the PR curves, while the second row shows the ROC curves. We observed that Splam outperformed SpliceAI on all three datasets, and its performance was substantially better than both SpliceAI and SpliceAI-10k-Ns in the flowering plant *Arabidopsis thaliana*. For *Arabidopsis*, the AUCPR values for Splam, SpliceAI, and SpliceAI-10k-Ns are 0.996, 0.990, and 0.9831, respectively; meanwhile, the AUROC values for Splam, SpliceAI, and SpliceAI-10k-Ns are 0.996, 0.987, and 0.978, respectively.

**Fig S10:** A joint scatterplot visualizing the relationship between intron lengths and score for Splam (green) and SpliceAI (orange) on the TAIR10 dataset. The left column displays intron lengths below 400nt, while the right column displays lengths above 400nt. The 400nt threshold reflects the minimum length necessary to avoid sequence overlap between the 200nt-flanked donor and acceptor sites for Splam. It additionally provides filtering of short introns for better comparison to the longer mammalian datasets. The top row shows donor site scores and the bottom row acceptor site scores. The rectangular histogram plots display the marginal distributions along each axis. The marginal score distributions do not significantly change from < 400nt to > 400nt for either model, indicating that TAIR10’s large discrepancy between Splam and SpliceAI scores cannot be simply attributed to shorter intron lengths.

**Fig S11:** Frequency distribution and kernel density plots of donor and acceptor site scores. The left column presents the donor site score distribution, and the right column shows the acceptor site score distribution for: **(a)** Novel-2, **(b)** Novel-20, **(c)** Novel-40, **(d)** Novel-60, **(e)** Novel-80, and **(f)** Novel-100.

**Fig S12:** Results of evaluating unannotated splice junctions across GTEx-supported datasets with varying spliced alignment counts. (a) Scatter plots depict the proportion of splice sites with Splam scores above 0.5 for a given depth of coverage. Donors are shown on left and acceptors on right. (b) Scatter plots display the mean Splam scores for both donor (left) and acceptor (right) sites compared to their respective spliced alignment counts. In each plot, the trend line is illustrated in red.

**Fig S13:** Performance of Splam with varying input sequence lengths across 15 epochs on the testing dataset, showing top-k accuracy, AUPRC (Area Under the Precision-Recall Curve), precision, and recall metrics for **(a)** donor and **(b)** acceptor sites. The blue curve represents the model trained with an input sequence length of 40 (Splam-40); the orange curve represents the model trained with an input sequence length of 100 (Splam-100); the green curve represents the model trained with an input sequence length of 200 (Splam-200); the red curve represents the model trained with an input sequence length of 400 (Splam-400); the purple curve represents the model trained with an input sequence length of 600 (Splam-600); and the brown curve represents the model trained with an input sequence length of 800 (Splam-800). A total of 9,000 steps were computed, with 600 steps following each epoch training update. The moving average with window size 40 was applied to all the curves.

**Fig S14:** Ablation study of the Splam model by sequential removal of residual groups. The AUPRC (Area Under the Precision-Recall Curve), precision, and recall metrics are shown for the testing dataset at **(a)** donor and **(b)** acceptor sites. The blue curve represents the model with one residual group, the orange curve represents two residual groups, the green curve represents three residual groups, the red curve represents four residual groups, and the purple curve represents five residual groups. A total of 9,000 steps were computed, with 600 steps following each epoch training update. The moving average with window size 40 was applied to all the curves.


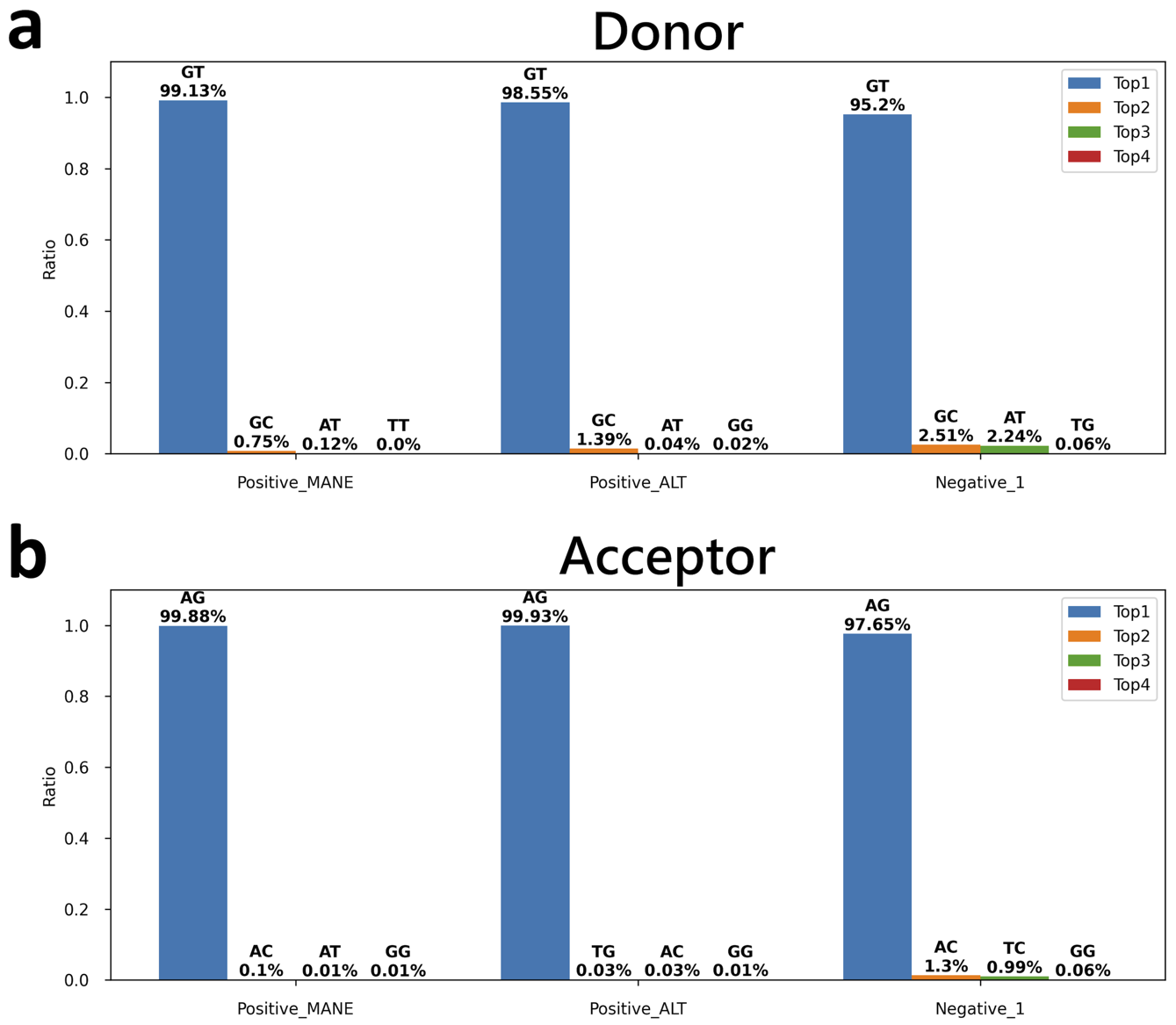


**Fig S15:** The four most frequently-occuring dinucleotides, by proportion, for the **(a)** donor and **(b)** acceptor sites for Positive-MANE, Positive-Alt, and Negative-1. It is observed that the canonical GT-AG donor-acceptor pair predominates across all three datasets with Negative-1 has the lowest frequency.

**Supplementary Tables**

| **Tools** | | **Accuracy (%)**  **(Donor / Acceptor / Junction)** | **Recall (%)**  **(Donor / Acceptor / Junction)** | **Precision (%)**  **(Donor / Acceptor / Junction)** |
| --- | --- | --- | --- | --- |
| *Pan troglodytes* | Splam | 97.6 / 97.6 / 97.5 | 95.3 / 95.4 / 95.1 | 99.9 / 99.8 / 99.9 |
|  | SpliceAI | 95.6 / 95.9 / 93.4 | 91.6 / 92.2 / 86.8 | 99.6 / 99.7 / 99.9 |
| *Mus musculus* | Splam | 96.9 / 96.9 / 96.7 | 94.0 / 94.0 / 93.6 | 99.8 / 99.8 / 99.8 |
|  | SpliceAI | 93.5 / 94.8 / 90.6 | 87.3 / 89.9 / 81.1 | 99.7 / 99.6 / 99.9 |
| *Arabidopsis thaliana* | Splam | 95.0 / 94.9 / 94.7 | 90.3 / 90.1 / 89.6 | 99.6 / 99.6 / 99.7 |
|  | SpliceAI | 87.0 / 84.4 / 78.6 | 74.3 / 69.6 / 57.3 | 99.6 / 98.9 / 99.9 |

**Table S1**: The accuracy, recall, and precision of donor sites, acceptor sites, and splice junctions at the score threshold of 0.1 for Splam and SpliceAI in chimpanzee (Pan troglodytes), mouse (Mus musculus), and the flowering plant Arabidopsis thaliana.

| **Sample** | **Precision** | | | **Recall** | | |
| --- | --- | --- | --- | --- | --- | --- |
|  | **Original (%)** | **Cleanup (%)** | $\boldsymbol{\Delta}$ **(%)** | **Original (%)** | **Cleanup (%)** | $\boldsymbol{\Delta}$ **(%)** |
| R2826 | 51.28 | 71.84 | 20.56 $\uparrow$ | 60.00 | 59.31 | ﻿-0.69 $\downarrow$ |
| R2835 | 56.01 | 74.51 | 18.50 $\uparrow$ | 58.80 | 58.16 | ﻿-0.64 $\downarrow$ |
| R2839 | 49.65 | 64.01 | 14.36 $\uparrow$ | 62.96 | 62.16 | ﻿-0.81 $\downarrow$ |
| R2845 | 54.78 | 74.02 | 19.24 $\uparrow$ | 58.48 | 57.85 | ﻿-0.63 $\downarrow$ |
| R2855 | 45.91 | 65.47 | 19.56 $\uparrow$ | 59.63 | 58.97 | ﻿-0.66 $\downarrow$ |
| R2857 | 51.69 | 70.86 | 19.17 $\uparrow$ | 57 29 | 56.72 | ﻿-0.57 $\downarrow$ |
| R2869 | 42.44 | 66.84 | 24.40 $\uparrow$ | 58.97 | 58.25 | ﻿-0.73 $\downarrow$ |
| R2874 | 36.68 | 61.59 | 24.91 $\uparrow$ | 61.18 | 60.40 | ﻿-0.78 $\downarrow$ |
| R2894 | 60.19 | 77.36 | 17.17 $\uparrow$ | 57.91 | 57.33 | ﻿-0.58 $\downarrow$ |
| R2895 | 50.70 | 71.73 | 21.03 $\uparrow$ | 60.23 | 59.53 | ﻿-0.70 $\downarrow$ |

**Table S2**: The precision and recall scores at the intron level for the 10 poly-A captured samples.

| **Sample** | **Precision** | | | **Recall** | | |
| --- | --- | --- | --- | --- | --- | --- |
|  | **Original (%)** | **Cleanup (%)** | $\boldsymbol{\Delta}$ **(%)** | **Original (%)** | **Cleanup (%)** | $\boldsymbol{\Delta}$ **(%)** |
| R12258 | 51.31 | 80.36 | 29.05 $\uparrow$ | 60.16 | 59.33 | ﻿-0.83 $\downarrow$ |
| R12260 | 37.92 | 74.45 | 36.53 $\uparrow$ | 63.23 | 62.16 | ﻿-1.07 $\downarrow$ |
| R12263 | 44.71 | 78.05 | 33.34 $\uparrow$ | 61.43 | 60.50 | ﻿-0.93 $\downarrow$ |
| R12265 | 45.74 | 79.75 | 34.02 $\uparrow$ | 60.03 | 59.15 | ﻿-0.88 $\downarrow$ |
| R12266 | 43.31 | 76.66 | 33.35 $\uparrow$ | 62.24 | 61 25 | ﻿-0.99 $\downarrow$ |
| R12277 | 53.07 | 85.11 | 32.04 $\uparrow$ | 57.06 | 56.33 | ﻿-0.73 $\downarrow$ |
| R12278 | 43.17 | 76.89 | 33.72 $\uparrow$ | 61 95 | 61.01 | ﻿-0.94 $\downarrow$ |
| R12280 | 40.93 | 74.99 | 34.06 $\uparrow$ | 62.77 | 61.80 | ﻿-0.98 $\downarrow$ |
| R12285 | 58.52 | 86.06 | 27.53 $\uparrow$ | 55.85 | 55.20 | ﻿-0.65 $\downarrow$ |
| R12287 | 39.36 | 73.19 | 33.83 $\uparrow$ | 63.75 | 62.68 | ﻿-1.07 $\downarrow$ |

**Table S3**: The precision and recall scores at the intron level for the 10 ribosomal RNA depletion samples.

| **Sample** | **Precision** | | | **Recall** | | |
| --- | --- | --- | --- | --- | --- | --- |
|  | **Original (%)** | **Cleanup (%)** | $\boldsymbol{\Delta}$ **(%)** | **Original (%)** | **Cleanup (%)** | $\boldsymbol{\Delta}$ **(%)** |
| R2826 | 39.2 | 41.6 | 2.4 $\uparrow$ | 10.6 | 10.6 | 0.0 |
| R2835 | 41.4 | 43.5 | 2.1 $\uparrow$ | 10.1 | 10.1 | 0.0 |
| R2839 | 37.7 | 40.3 | 2.6 $\uparrow$ | 11.1 | 11.1 | 0.0 |
| R2845 | 38.8 | 40.9 | 2.1 $\uparrow$ | 10.1 | 10.1 | 0.0 |
| R2855 | 39.4 | 41.8 | 2.4 $\uparrow$ | 10.4 | 10.4 | 0.0 |
| R2857 | 38.8 | 41.0 | 2.2 $\uparrow$ | 95 | 9.5 | 0.0 |
| R2869 | 37.9 | 40.4 | 2.5 $\uparrow$ | 10.1 | 10.1 | 0.0 |
| R2874 | 36.6 | 39 3 | 2.7 $\uparrow$ | 10.9 | 10.9 | 0.0 |
| R2894 | 40.5 | 42.8 | 2.3 $\uparrow$ | 9.9 | 9.9 | 0.0 |
| R2895 | 40.7 | 43.0 | 2.3 $\uparrow$ | 10.8 | 10.8 | 0.0 |

**Table S4**: The precision and recall scores at the transcript level for the 10 poly-A captured samples.

| **Sample** | **Precision** | | | **Recall** | | |
| --- | --- | --- | --- | --- | --- | --- |
|  | **Original (%)** | **Cleanup (%)** | $\boldsymbol{\Delta}$ **(%)** | **Original (%)** | **Cleanup (%)** | $\boldsymbol{\Delta}$ **(%)** |
| R12258 | 30.0 | 36.1 | 6.1 $\uparrow$ | 9.2 | 9.4 | 0.2 $\uparrow$ |
| R12260 | 24.7 | 31.0 | 6.3 $\uparrow$ | 9.5 | 9.9 | 0.4 $\uparrow$ |
| R12263 | 27 1 | 33.1 | 6.0 $\uparrow$ | 9.4 | 9.6 | 0.2 $\uparrow$ |
| R12265 | 25.9 | 31.9 | 6.0 $\uparrow$ | 8.7 | 8.9 | 0.2 $\uparrow$ |
| R12266 | 27.1 | 32.9 | 5.8$\uparrow$ | 9.7 | 9.9 | 0.2 $\uparrow$ |
| R12277 | 25.9 | 31.2 | 5.3 $\uparrow$ | 7.4 | 7.6 | 0.2 $\uparrow$ |
| R12278 | 27.9 | 34 4 | 6.5 $\uparrow$ | 97 | 10.0 | 0.3 $\uparrow$ |
| R12280 | 27.0 | 33.4 | 6.4 $\uparrow$ | 9.9 | 10.2 | 0.3 $\uparrow$ |
| R12285 | 26.0 | 32 2 | 6.2 $\uparrow$ | 7.1 | 73 | 0.2 $\uparrow$ |
| R12287 | 26.0 | 31.5 | 5.5 $\uparrow$ | 9.9 | 10.2 | 0.3 $\uparrow$ |

**Table S5**: The precision and recall scores at the transcript level for the 10 ribosomal RNA depletion samples.

| **Sample** | **Matching intron chains** | | | **Matching transcripts** | | |
| --- | --- | --- | --- | --- | --- | --- |
|  | **Original** | **Cleanup** | $\boldsymbol{\Delta}$ **(%)** | **Original** | **Cleanup** | $\boldsymbol{\Delta}$ **(%)** |
| R2826 | 21,594 | 21,596 | 0.01$\uparrow$ | 21,858 | 21,861 | 0.02 $\uparrow$ |
| R2835 | 20,697 | 20,647 | ﻿-0.24 $\downarrow$ | 20,958 | 20,915 | ﻿-0.21 $\downarrow$ |
| R2839 | 22,801 | 22,789 | ﻿-0.05 $\downarrow$ | 23,024 | 23,012 | ﻿-0.05 $\downarrow$ |
| R2845 | 20,612 | 20,602 | ﻿-0.05 $\downarrow$ | 20,875 | 20,870 | ﻿-0.02 $\downarrow$ |
| R2855 | 21,291 | 21,262 | -﻿0.14 $\downarrow$ | 21,526 | 21,508 | ﻿-0.08 $\downarrow$ |
| R2857 | 19,393 | 19,364 | -﻿0.15 $\downarrow$ | 19,629 | 19,596 | ﻿-0.17 $\downarrow$ |
| R2869 | 20,532 | 20,493 | ﻿-0.19 $\downarrow$ | 20,835 | 20,810 | ﻿-0.10 $\downarrow$ |
| R2874 | 22,187 | 22,180 | ﻿-0.03 $\downarrow$ | 22,478 | 22,484 | 0.03 $\uparrow$ |
| R2894 | 20,236 | 20,265 | 0.14 $\uparrow$ | 20,481 | 20,514 | 0.16 $\uparrow$ |
| R2895 | 2,157 | 22,105 | -0.23 $\downarrow$ | 22,420 | 22,369 | -0.23 $\downarrow$ |

**Table S6**: The number of matching intron chains and transcripts for the 10 poly-A captured samples.

| **Sample** | **Matching intron chains** | | | **Matching transcripts** | | |
| --- | --- | --- | --- | --- | --- | --- |
|  | **Original** | **Cleanup** | $\boldsymbol{\Delta}$ **(%)** | **Original** | **Cleanup** | $\boldsymbol{\Delta}$ **(%)** |
| R12258 | 18,869 | 19,267 | 2.1 $\uparrow$ | 19,071 | 19,500 | 2.3 $\uparrow$ |
| R12260 | 19,562 | 20,285 | 3.7 $\uparrow$ | 19,752 | 20,513 | 3.8 $\uparrow$ |
| R12263 | 19,207 | 19,661 | 2.4 $\uparrow$ | 19,419 | 19,910 | 2.5 $\uparrow$ |
| R12265 | 17,799 | 18,291 | 2.8 $\uparrow$ | 17,995 | 18,513 | 2.9 $\uparrow$ |
| R12266 | 19,838 | 20,281 | 2.3 $\uparrow$ | 20,040 | 20,525 | 2.4 $\uparrow$ |
| R12277 | 15,198 | 15,439 | 1.6 $\uparrow$ | 15,384 | 15,656 | 1.8 $\uparrow$ |
| R12278 | 19,835 | 20,359 | 2.4 $\uparrow$ | 20,063 | 20,624 | 2.8 $\uparrow$ |
| R12280 | 20,293 | 20,868 | 2.9 $\uparrow$ | 20,504 | 21,114 | 3.0 $\uparrow$ |
| R12285 | 14,615 | 14,971 | 2.4 $\uparrow$ | 14,774 | 15,147 | 2.5 $\uparrow$ |
| R12287 | 20,331 | 20,796 | 2.3 $\uparrow$ | 20,532 | 21,032 | 2.4 $\uparrow$ |

**Table S7**: The number of matching intron chains and transcripts for the 10 ribosomal RNA depletion samples.

| **Sample** | **Hypothetical exons** | | | **Missed exons** | | |
| --- | --- | --- | --- | --- | --- | --- |
|  | **Original (%)** | **Cleanup (%)** | $\boldsymbol{\Delta}$ **(%)** | **Original (%)** | **Cleanup (%)** | $\boldsymbol{\Delta}$ **(%)** |
| R2826 | 7.9 | 5.7 | ﻿-2.2 $\downarrow$ | 47.7 | 47 9 | 0.2 $\uparrow$ |
| R2835 | 6.6 | 4.7 | ﻿-1.9 $\downarrow$ | 49.0 | 49.2 | 0.2 $\uparrow$ |
| R2839 | 9.7 | 6.8 | ﻿-2.9 $\downarrow$ | 45.4 | 45.7 | 0.3 $\uparrow$ |
| R2845 | 7.5 | 5.3 | ﻿-2.2 $\downarrow$ | 48.9 | 49.1 | 0.2 $\uparrow$ |
| R2855 | 7.3 | 5.0 | ﻿-2.3 $\downarrow$ | 48.3 | 48.5 | 0.2 $\uparrow$ |
| R2857 | 6.4 | 4.3 | ﻿-2.1 $\downarrow$ | 50.1 | 50.3 | 0.2 $\uparrow$ |
| R2869 | 7.7 | 5.2 | ﻿-2.5 $\downarrow$ | 48.7 | 49.0 | 0.3 $\uparrow$ |
| R2874 | 9.4 | 6.6 | ﻿-2.8 $\downarrow$ | 46.7 | 46.9 | 0.2 $\uparrow$ |
| R2894 | 6.6 | 4.7 | -﻿1.9 $\downarrow$ | 49 4 | 49.7 | 0.3 $\uparrow$ |
| R2895 | 7.6 | 5.5 | ﻿-2.1 $\downarrow$ | 47.8 | 48.1 | 0.3 $\uparrow$ |

**Table S8**: The percentage of hypothetical exons and missed exons for the 10 poly-A captured samples.

| **Sample** | **Hypothetical exons** | | | **Missed exons** | | |
| --- | --- | --- | --- | --- | --- | --- |
|  | **Original (%)** | **Cleanup (%)** | $\boldsymbol{\Delta}$ **(%)** | **Original (%)** | **Cleanup (%)** | $\boldsymbol{\Delta}$ **(%)** |
| R12258 | 12.9 | 6.4 | -6.5 $\downarrow$ | 47.1 | 47.4 | 0.3 $\uparrow$ |
| R12260 | 18.0 | 9.1 | -8.9 $\downarrow$ | 44.6 | 45.0 | 0.4 $\uparrow$ |
| R12263 | 15.2 | 7.5 | -﻿7.7 $\downarrow$ | 46.1 | 46.5 | 0.4 $\uparrow$ |
| R12265 | 15.3 | 7.2 | -﻿8.1 $\downarrow$ | 47.3 | 47 6 | 0.3 $\uparrow$ |
| R12266 | 15.9 | 8.3 | ﻿-7.6 $\downarrow$ | 45.5 | 45.9 | 0.4 $\uparrow$ |
| R12277 | 12.5 | 5.4 | ﻿-7.1 $\downarrow$ | 49.6 | 49.9 | 0.3 $\uparrow$ |
| R12278 | 15.2 | 7.6 | ﻿-7.6 $\downarrow$ | 45.8 | 46.1 | 0.3 $\uparrow$ |
| R12280 | 16.8 | 8.6 | ﻿-8.2 $\downarrow$ | 45.1 | 45.5 | 0.4 $\uparrow$ |
| R12285 | 12.5 | 5.1 | -7.4 $\downarrow$ | 50.7 | 50.9 | 0.2 $\uparrow$ |
| R12287 | 16.8 | 9.2 | -7.6 $\downarrow$ | 44.3 | 44.7 | 0.4 $\uparrow$ |

**Table S9**: The precision of hypothetical exons and missed exons for the 10 ribosomal RNA depletion samples.

| **Sample** | **Hypothetical introns** | | | **Missed introns** | | |
| --- | --- | --- | --- | --- | --- | --- |
|  | **Original (%)** | **Cleanup (%)** | $\boldsymbol{\Delta}$ **(%)** | **Original (%)** | **Cleanup (%)** | $\boldsymbol{\Delta}$ **(%)** |
| R2826 | 4.2 | 2.5 | ﻿-1.7 $\downarrow$ | 35.9 | 36.3 | 0.4 $\uparrow$ |
| R2835 | 3.5 | 2.1 | ﻿-1.4 $\downarrow$ | 37.4 | 37.6 | 0.2 $\uparrow$ |
| R2839 | 5.3 | 3.2 | ﻿-2.1 $\downarrow$ | 33.6 | 34.0 | 0.4 $\uparrow$ |
| R2845 | 3.9 | 2.3 | ﻿-1.6 $\downarrow$ | 37.4 | 37 7 | 0.3 $\uparrow$ |
| R2855 | 3.9 | 2.2 | ﻿-1.7 $\downarrow$ | 36.4 | 36.7 | 0.3 $\uparrow$ |
| R2857 | 3.3 | 1.8 | ﻿-1.5 $\downarrow$ | 38.5 | 38.8 | 0.3 $\uparrow$ |
| R2869 | 4.1 | 2.2 | ﻿-1.9 $\downarrow$ | 37 5 | 37.8 | 0.3 $\uparrow$ |
| R2874 | 5.0 | 2.9 | -2.1 $\downarrow$ | 35.0 | 35.3 | 0.3 $\uparrow$ |
| R2894 | 3.4 | 2.0 | -1.4 $\downarrow$ | 37.9 | 38.2 | 0.3 $\uparrow$ |
| R2895 | 4.0 | 2.4 | -1.6 $\downarrow$ | 35.9 | 36.2 | 0.3 $\uparrow$ |

**Table S10**: The percentage of hypothetical introns and missed intron for the 10 poly-A captured samples.

| **Sample** | **Hypothetical introns** | | | **Missed introns** | | |
| --- | --- | --- | --- | --- | --- | --- |
|  | **Original (%)** | **Cleanup (%)** | $\boldsymbol{\Delta}$ **(%)** | **Original (%)** | **Cleanup (%)** | $\boldsymbol{\Delta}$ **(%)** |
| R12258 | 7.6 | 2.9 | ﻿-4.7 $\downarrow$ | 35.7 | 36.1 | 0.4 $\uparrow$ |
| R12260 | 10 5 | 3.9 | ﻿-6.6 $\downarrow$ | 33.8 | 34.0 | 0.2 $\uparrow$ |
| R12263 | 8.9 | 3.3 | ﻿-5.6 $\downarrow$ | 35.0 | 35.4 | 0.4 $\uparrow$ |
| R12265 | 8.9 | 3.0 | ﻿-5.9 $\downarrow$ | 36.4 | 36.6 | 0.2 $\uparrow$ |
| R12266 | 9.3 | 3.6 | -﻿5.7 $\downarrow$ | 34.3 | 34 7 | 0.4 $\uparrow$ |
| R12277 | 7.2 | 2.3 | ﻿-4.9 $\downarrow$ | 38.8 | 39.1 | 0.3 $\uparrow$ |
| R12278 | 9.0 | 3.5 | ﻿-5.5 $\downarrow$ | 34.4 | 34.8 | 0.4 $\uparrow$ |
| R12280 | 9.8 | 3.8 | ﻿-6.0 $\downarrow$ | 33.8 | 34.2 | 0.4 $\uparrow$ |
| R12285 | 7.4 | 2.1 | ﻿-5.3 $\downarrow$ | 39.9 | 40.1 | 0.2 $\uparrow$ |
| R12287 | 9.9 | 4.1 | ﻿-5.8 $\downarrow$ | 33.1 | 33.4 | 0.3 $\uparrow$ |

**Table S11**: The precision of hypothetical introns and missed introns for the 10 ribosomal RNA depletion samples.

| **Positive_MANE** | Donor | **GT** | **GC** | **AT** | **TT** | **GA** |
| --- | --- | --- | --- | --- | --- | --- |
|  |  | 178618 | 1346 | 209 | 8 | 8 |
|  | Acceptor | **AG** | **AC** | **AT** | **GG** | **AA** |
|  |  | 178874 | 184 | 14 | 10 | 9 |
| **Positive_Alt** | Donor | **GT** | **GC** | **AT** | **GG** | **GA** |
|  |  | 84633 | 1195 | 36 | 17 | 13 |
|  | Acceptor | **AG** | **TG** | **AC** | **GG** | **AT** |
|  |  | 85815 | 25 | 24 | 12 | 9 |

**Table S12**: The count of each dinucleotide for both the donor and acceptor sites in the Positive-MANE and Positive-Alt datasets.

1. Varabyou, A. et al. CHESS 3: an improved, comprehensive catalog of human genes and transcripts based on large-scale expression data, phylogenetic analysis, and protein structure. *Genome Biology* **24**, 1-16 (2023).
