# Supplementary material for: Splam: a deep-learning-based splice site predictor that improves spliced alignments
Source: Genome Biol. 2024 Sep 16;25:243. doi: 10.1186/s13059-024-03379-4 (PMC11406845; doi:10.1186/s13059-024-03379-4)
Supplement: Supplementary file 2 — Additional file 2: Review history. [file 13059_2024_3379_MOESM2_ESM.docx]

Review History

**First round of review**

**Reviewer 1**

**Were you able to assess all statistics in the manuscript, including the appropriateness of statistical tests used?**

Yes: The results sections provided all the necessary statistics.

**Were you able to directly test the methods?**

No.

**Comments to author:**

The authors introduce Splam, a deep residual convolutional neural network designed for splice site prediction capable of generalizing across species to identify both donor and acceptor sites simultaneously. The input format is clearly elucidated in Figures 1c and 1d, highlighting a key strength of Splam. Additionally, the tool is accompanied by comprehensive documentation, enhancing its usability and accessibility.

*While the architecture of Splam is rooted in convolutional neural networks, recent advancements in deep learning have introduced several tools like EnsembleSplice, Splice2Deep, and SpliceBERT, which employ more sophisticated techniques. These models, such as the bidirectional encoder representations from transformers in SpliceBERT (https://doi.org/10.1101/2023.01.31.526427), offer alternative approaches to solving similar problems. Presenting them alongside Splam provides readers with a comprehensive comparison rather than solely focusing on Splice Ai.

*Could the authors conduct an ablation study to determine whether the robustness of the method is rooted in the input formatting of 800 nt, including both donor and acceptor sites, or is a result of the architecture?

*The authors did not specify their rationale for choosing one-hot encoding over distributed representation (dense vector encoding), an NLP technique, in Section 5.2. It's worth considering whether utilizing distributed representation could have potentially improved performance by capturing sequence information more effectively.

*Although the authors have provided a GitHub repository containing the code, it was challenging to locate the datasets utilized in their study. It is imperative that the authors archive the datasets employed for training purposes, ensuring they are readily accessible in a repository and distinctly labeled as training and testing datasets.

**Reviewer 2**

**Were you able to assess all statistics in the manuscript, including the appropriateness of statistical tests used?**

Yes: The evaluation of the approach does not rely on especially sophisticated statistics, although I have some concerns about the evaluation methodology as explained in the full review.

**Were you able to directly test the methods?**

Yes.

**Comments to author:**

The paper introduces Splam, a new deep learning approach to recognize splice sites from genomic sequences.

It's main claim is that it is able to improve upon the accuracy of SpliceAI while using a much shorter flanking context.

A big aspect of novelty is the fact that it evaluates donor and acceptor sites in pairs, and that it is trained on multiple isoforms, so it is able to identify alternate splicing.

Splice AI takes as input a single window around a candidate splice site and its output is a classification of that site as either an acceptor or a donor. This means that for a gene of length L, identifying the splice sites takes O(L).

From what I understand, this approach actually involves two windows which are fed as input to the network, where one window is the acceptor window (400nt wide) and the other is the donor window (400nt wide), and the network takes all 800nt as input and produces a prediction on whether they are matching acceptor and donor. So Identifying the splice sites for a gene of size L takes O(L^2). This was at least my understanding of the approach, it would be good if the paper described this more explicitly.

Related to this point, one of the features that is heavily emphasized by the paper is that the scores of the acceptor and donor site are usually very consistent. This is emphasized in figure 2c-d, as well as in figure 3a. This seems like an important benefit of Splam, but it begs the question of why does the model produce separate scores for acceptor and donor given that the model evaluates acceptor and donor sites as a pair and those values are strongly correlated anyway.

The paper also addresses another important shortcoming of SpliceAI which is that output values tend to cluster around zero, even for true splice sites, so the approach is highly sensitive to the choice of threshold. In contrast, the output values in Splam cluster more heavily around 1 and zero, making them a lot less sensitive on the choice of threshold.

The paper also shows that Splam generalizes much better to different species, including species as far removed from humans as Arabidopsis thaliana.

I feel less qualified to evaluate the proposed application of filtering spliced alignments on transcriptome assembly.

Questions and concerns.

For me, one of the biggest questions I had in evaluating this work was the choice of negative splice junctions. The authors make a strong attempt to avoid including pairs that may actually be spliced, but this risks excluding from the negative datasets more ambiguous pairs. I was especially concerned by Figure 6b, because it seems to suggest that the positive and negative sites look quite different for both acceptors and donnors. Is this a misreading of Figure 6?

I would really like to see more evidence that the strong results in the paper are not an artifact of the conservative choice of negative datasets. The worry is that there are many acceptor donor pairs that in practice have very little chance of marking an intron but are excluded from the negative sets and therefore make the false positive rate seem lower than it really is.

One problem is in the strong distinction that this model makes between positive and negative pairs, when in practice there are a lot of pairs with varying levels of evidence for whether or not they bound a possible intron. One experiment I would really like to see is one where you take all possible pairs of acceptor and donor sites within a set of genes, and then you assign a score based on the evidence you have that this pair represents an intron. So all the pairs that are part of your negative dataset would be close to zero, but many other pairs would have scores in between zero and one. Then I would like to see a plot that has this score in the x-axis and the output produced by your model in the y-axis.

Smaller issues.

The manuscript seems to be missing a supplement. There are many different references to figures S1, S2, but I did not see them.

There is also a reference to a "Figure 3c-d" but there is no figure 3 (d) in the paper.

**Authors Response**

**Point-by-point responses to the reviewers’ comments:**

**Reviewer #1:**

The authors introduce Splam, a deep residual convolutional neural network designed for splice site prediction capable of generalizing across species to identify both donor and acceptor sites simultaneously. The input format is clearly elucidated in Figures 1c and 1d, highlighting a key strength of Splam. Additionally, the tool is accompanied by comprehensive documentation, enhancing its usability and accessibility.

*Thanks for the positive feedback about Splam.*

**1.1** While the architecture of Splam is rooted in convolutional neural networks, recent advancements in deep learning have introduced several tools like EnsembleSplice, Splice2Deep, and SpliceBERT, which employ more sophisticated techniques. These models, such as the bidirectional encoder representations from transformers in SpliceBERT (<https://doi.org/10.1101/2023.01.31.526427>), offer alternative approaches to solving similar problems. Presenting them alongside Splam provides readers with a comprehensive comparison rather than solely focusing on SpliceAI.

***=== Response 1.1 begin ===***

*In response to the reviewer's suggestion, we benchmarked six different splice site predictors. In particular, we tried to evaluate SpliceAI ^1^, Pangolin ^2^, Spliceator ^3^, SpliceBERT ^4^, EnsembleSplice ^5^, and Splice2Deep ^6^; however we were only able to successfully run SpliceAI, Pangolin and Spliceator. We have now updated the manuscript to include comparisons to these three systems, now described in* ***Note S1*** *and* ***Figure S1****.*

*Notes on the problems we encountered with the other three systems: first, we were unable to find a pretrained SpliceBERT model on GitHub (*[*https://github.com/biomed-AI/SpliceBERT*](https://github.com/biomed-AI/SpliceBERT)*). Therefore, we attempted to fine-tune the model using the AutoModelForSequenceClassification function, following the provided documentation, but this produced error messages which we were unable to debug. Second, we attempted to install EnsembleSplice (*[*https://github.com/OluwadareLab/EnsembleSplice*](https://github.com/OluwadareLab/EnsembleSplice)*) using the recommended requirements.txt file, but this attempt failed. Third, we were able to install Splice2Deep (*[*https://github.com/SomayahAlbaradei/Splice_Deep*](https://github.com/SomayahAlbaradei/Splice_Deep)*); however, we encountered errors when running Splice2Deep’s “Splice_Deep_Donor.py” and “Splice_Deep_Acceptor.py” scripts.*

*The updated paragraph in the Results section is as follows:*

*“... Although our discussion below focuses on comparisons against SpliceAI, we also benchmarked Splam against other neural-network-based splice site predictors, described in Supplementary Note S1 and Figure S1. SpliceAI and Splam both outperformed these competing methods.”*

*The supplementary files now include the following additional results:*

*```````````````````````````````````````````*

***Note S1.*** *Results of benchmarking Splam to other splice site predictors*

*We benchmarked Splam against SpliceAI ^1^, Pangolin ^2^, and Spliceator ^7^. To evaluate all tools, we compiled a test set consisting of 1,000 splice junctions each from the Positive-MANE and Positive-Alt datasets, along with 2,000 junctions from the Negative-1 dataset, totaling 4,000 splice junctions. This dataset included junctions only from chromosomes 1 and 9, which were excluded from training for all tools.*

*We ran SpliceAI with the same settings as described in the main paper. SpliceAI's output includes probabilities that indicate whether a position in the input sequence is a donor, acceptor, or non-splice site. We determined the final donor and acceptor scores for each splice junction by computing the average probabilities that were obtained from five SpliceAI model runs. Pangolin produces four predictions for four different specific tissue types; therefore we average these four predictions in order to compare Pangolin to the other systems. We ran Spliceator with a window size of 400 nt in order to match Splam’s input size.*

*We compared the performance of Splam, SpliceAI, Pangolin, and Spliceator using precision-recall curves (Figure S1). For both the donor and acceptor site predictions, Splam achieved an AUC of 0.99 and, while SpliceAI achieved an AUC of 0.98. Spliceator achieved an AUC of 0.85 for the donor site and an AUC of 0.59 for the acceptor site, while Pangolin achieved AUCs of 0.77 and 0.82 for the donor and acceptor sites, respectively. Based on these results, we focused on comparisons with SpliceAI for the remainder of our analysis.*

***Figure S1:*** *Precision-recall curves comparing Splam (blue curve), SpliceAI (orange curve), Spliceator (green curve), and Pangolin (red curve) for donor (a) and acceptor (b) splice site predictions. The analysis was performed on a test dataset comprising 2,000 positive and 2,000 negative splice junctions.*

***=== Response 1.1 end ===***

**1.2** Could the authors conduct an ablation study to determine whether the robustness of the method is rooted in the input formatting of 800nt, including both donor and acceptor sites, or is a result of the architecture?

***=== Response 1.2 begin ===***

*We conducted several new experiments in response to this request. First, we retrained Splam with different lengths of input sequences — 40 bp, 100 bp, 200 bp, 400 bp, 600 bp, and 800 bp — and describe those experiments in* ***Note S3*** *and* ***Figure S13*** *in the supplementary files.*

*We also conducted an ablation study on the Splam model architecture, now described in* ***Note S4*** *and* ***Figures S14*** *in the supplementary files.*

*Additionally, we have added a paragraph to the Discussion section to summarize these experiments, as follows:*

*“We conducted two sets of experiments to evaluate some key parameters in Splam's design. In the first set, we trained the model on input sequences ranging from 40 to 800 nucleotides (nt), as described in Supplementary Note S3 and Figure S13. Those experiments led us to choose 800nt as the input for our final model. In the second experiment, we conducted an ablation study to determine the optimal number of residual groups within the neural network, varying this number from one to five. That experiment, described in Supplementary Note S4 and Figure S14, led us to choose five residual groups for the model’s configuration.”*

*Below we included the text of the two new supplementary notes, and the two new figures, describing these experiments:*

*```````````````````````````````````````````*

***Note S3.*** *Training Splam across various input sequence lengths*

*In this study, we introduced a novel approach where the system learns to recognize paired donor and acceptor sites, using windows of 400 nt centered on each site. We concatenated these pairs to form 800 nt input sequences for training Splam.*

*In this experiment, our goal was to understand how the input window size influences performance. Consequently, we varied the input sequence lengths to 40, 100, 200, 400, 600, and 800 nucleotides, and trained a separate Splam model on each. We designated the models trained with different sequence lengths as Splam-40, Splam-100, Splam-200, Splam-400, Splam-600, and Splam-800, respectively.*

*For the model training, we randomly selected 20,000 splice junctions from each of the Positive-MANE and Positive-Alt, as well as 40,000 splice junctions from each of the Negative-Random and Negative-1. The training dataset includes splice junctions from all chromosomes, except Chromosome 1 and 9. Similarly, for testing the model, we randomly selected 20,000 splice junctions from each of the Positive-MANE and Positive-Alt, along with 40,000 splice junctions from each of the Negative-Random and Negative-1. The testing dataset exclusively contains splice sites located on Chromosome 1 and 9. We used the Splam model architecture with five residual groups (Figure 1).*

*Each Splam model was trained for 15 epochs with a batch size of 100. The testing dataset, comprising 60,000 splice sites, was evaluated after each epoch, with each epoch consisting of 600 steps, resulting in a total of 9,000 steps. At each step, we calculated the AUPRC (Area Under the Precision-Recall Curve), precision, and recall metrics for both donor (Figure S13a) and acceptor sites (Figure S13b). We then applied a moving average with a window size of 40 to each metric, utilizing NumPy's convolution function for smoothing. This approach helps to observe the trend more clearly.*

*We observed that the model took longer to recognize splice sites with increased input sequence length, requiring additional update steps to process the greater volume of information and learn the correct patterns. After 15 epochs of training, the performances of the Splam-200, Splam-400, Splam-600, and Splam-800 models converged to similar levels (Figure S13). In contrast, the Splam-100 and Splam-40 models exhibited slightly lower top-k accuracy, AUPRC, and precision. This suggests that at least a 200-nucleotide flanking sequence is needed for accurate performance. We selected Splam-800 as the final model because it encompasses the most information, but further research is needed to determine whether a smaller window size might be superior.*

**Figure S13:** Performance of Splam with varying input sequence lengths across 15 epochs on the testing dataset, showing top-k accuracy, AUPRC (Area Under the Precision-Recall Curve), precision, and recall metrics for (a) donor and (b) acceptor sites. The blue curve represents the model trained with an input sequence length of 40 (Splam-40); the orange curve represents the model trained with an input sequence length of 100 (Splam-100); the green curve represents the model trained with an input sequence length of 200 (Splam-200); the red curve represents the model trained with an input sequence length of 400 (Splam-400); the purple curve represents the model trained with an input sequence length of 600 (Splam-600); and the brown curve represents the model trained with an input sequence length of 800 (Splam-800). A total of 9,000 steps were computed, with 600 steps following each epoch training update. The moving average with window size 40 was applied to all the curves.

*```````````````````````````````````````````*

***Note S4.*** *Ablation study of the Splam model architecture*

*To evaluate the robustness of the Splam model architecture, we conducted an ablation study by sequentially removing residual groups to observe the impact on performance across training and testing datasets. The Splam model comprises five residual groups, with each group containing four residual units. In our experiments, 'RSG_X' designates a configuration with X residual groups. Thus, the original model is designated 'RSG_5'.*

*For training the models, we randomly selected 20,000 splice junctions each from the Positive-MANE and Positive-Alt datasets, and 40,000 splice junctions each from the Negative-Random and Negative-1 datasets. The training dataset includes splice junctions from all chromosomes, except Chromosome 1 and 9. Similarly, for testing we randomly selected 20,000 splice junctions each from Positive-MANE and Positive-Alt, and 40,000 splice junctions each from Negative-Random and Negative-1. The testing dataset exclusively contains splice sites located on Chromosome 1 and 9.*

*In our experiments, we varied the number of residual groups between one and five. Each Splam model was trained for 15 epochs with a batch size of 100. The testing dataset, comprising 60,000 splice sites, was evaluated after each epoch, with each epoch consisting of 600 steps, resulting in a total of 9,000 steps. At each step, we calculated the AUPRC (Area Under the Precision-Recall Curve), precision, and recall metrics for both donor (Figure S14a) and acceptor sites (Figure S14b). We then applied a moving average with a window size of 40 to each metric, utilizing NumPy's convolution function for smoothing. This approach helps to observe the trend more clearly.*

*We observed that as the number of residual groups increased, the model's performance improved, as expected. The performance of RSG_4 (represented by the red curve) and RSG_5 (represented by the purple curve) was similar, suggesting that at least four residual groups are required for optimal accuracy. We chose the 5-residual group model architecture as the final model.*

**Figure S14:** Ablation study of the Splam model by sequential removal of residual groups. The AUPRC (Area Under the Precision-Recall Curve), precision, and recall metrics are shown for the testing dataset at (a) donor and (b) acceptor sites. The blue curve represents the model with one residual group, the orange curve represents two residual groups, the green curve represents three residual groups, the red curve represents four residual groups, and the purple curve represents five residual groups. A total of 9,000 steps were computed, with 600 steps following each epoch training update. The moving average with window size 40 was applied to all the curves.

***=== Response 1.2 end ===***

**1.3** The authors did not specify their rationale for choosing one-hot encoding over distributed representation (dense vector encoding), an NLP technique, in Section 5.2. It's worth considering whether utilizing distributed representation could have potentially improved performance by capturing sequence information more effectively.

***=== Response 1.3 begin ===***

*Our decision to use one-hot encoding instead of a distributed representation (dense vector encoding) was primarily guided by model simplicity, and also by its widespread use in similar tools: one-hot encoding is used in SpliceAI ^1^, Basenji ^8^, Spliceator ^3^, SpliceFinder ^9^, and Enformer ^10^. While we appreciate the reviewer's suggestion, trying out an entirely new input representation is beyond the scope of the current paper. We added a new sentence in the manuscript, in the Methods section, to explain our choice of representation. The updated text reads:*

*“The input sequences are then one-hot encoded, with* $[A, C,G,T,N]$ *represented as* $[1, 0, 0, 0]$*,* $[0, 1, 0, 0]$*,* $[0, 0, 1, 0]$*,* $[0, 0, 0, 1]$*, and* $[0, 0, 0, 0]$*. One-hot encoding was chosen for its simplicity and because it is widely used in similar applications, including SpliceAI, Basenji, Spliceator, SpliceFinder, and Enformer.”*

***=== Response 1.3 end ===***

**1.4** Although the authors have provided a GitHub repository containing the code, it was challenging to locate the datasets utilized in their study. It is imperative that the authors archive the datasets employed for training purposes, ensuring they are readily accessible in a repository and distinctly labeled as training and testing datasets.

***=== Response 1.4 begin ===***

*We have now archived the datasets used for training and testing Splam in Zenodo. Each dataset is distinctly labeled as training or testing, accompanied by detailed metadata explaining the dataset's origin, composition, and any preprocessing steps applied. We have provided a direct link to these datasets in the revised manuscript and in our project's GitHub repository, to ensure that readers of our paper can easily access and utilize these resources. We also added a sentence in the manuscript under “Availability of data and materials” as follows:*

*“All the training and testing dataset used in this study can be accessed in Zenodo at* [*https://doi.org/10.5281/zenodo.10957161*](https://doi.org/10.5281/zenodo.10957161)*.”*

***=== Response 1.4 end ===***

**Reviewer #2:**

**2.1** The paper introduces Splam, a new deep learning approach to recognize splice sites from genomic sequences. Its main claim is that it is able to improve upon the accuracy of SpliceAI while using a much shorter flanking context. A big aspect of novelty is the fact that it evaluates donor and acceptor sites in pairs, and that it is trained on multiple isoforms, so it is able to identify alternate splicing. Splice AI takes as input a single window around a candidate splice site and its output is a classification of that site as either an acceptor or a donor. This means that for a gene of length L, identifying the splice sites takes O(L). From what I understand, this approach actually involves two windows which are fed as input to the network, where one window is the acceptor window (400nt wide) and the other is the donor window (400nt wide), and the network takes all 800nt as input and produces a prediction on whether they are matching acceptor and donor. So identifying the splice sites for a gene of size L takes O(L^2). This was at least my understanding of the approach, it would be good if the paper described this more explicitly.

***=== Response 2.1 begin ===***

*We thank the referee for this thoughtful feedback. Regarding the refereee's concerns about computational efficiency, we should emphasize that Splam's intended application is not to perform an exhaustive search for every potential donor-acceptor pair within a gene, which would indeed result in* $O(L^{2})$ *complexity. Instead, Splam is intended for scenarios where there is prior information suggesting that a splice junction is present, either from RNA-Seq data or genomic annotations. Splam can analyze these junctions in linear time. We have added a paragraph in the Discussion section to clarify, as follows:*

*“Unlike previous systems, Splam analyzes donor and acceptor sites jointly, to capture dependencies between splice sites. This dual-window approach is not intended for exhaustive scanning across entire chromosomes, which might require considering a quadratic number of donor-acceptor site candidates. Instead, Splam is designed for evaluating putative splice junctions that have supporting evidence either from alignments of RNA-seq data or from existing annotation.”*

***=== Response 2.1 end ===***

**2.2** Related to this point, one of the features that is heavily emphasized by the paper is that the scores of the acceptor and donor site are usually very consistent. This is emphasized in figure 2c-d, as well as in figure 3a. This seems like an important benefit of Splam, but it begs the question of why does the model produce separate scores for acceptor and donor given that the model evaluates acceptor and donor sites as a pair and those values are strongly correlated anyway.

***=== Response 2.2 begin ===***

*In response to this question, we have added a paragraph to the Discussion section.*

*Despite the strong correlation between these scores, the model is deliberately engineered to generate separate scores for each site. This design can be valuable in scenarios where the scores diverge (although they usually are highly correlated, as the reviewer notes), suggesting that one of the sites might be weaker than the other.*

*While donor and acceptor site scores are usually consistent, there are many instances, some of which can be seen in Figure S3, where one site gets a high score while the other one gets a low or average score. These splice junctions might deserve further scrutiny, which would not be possible if we presented a single, combined score. In addition, providing separate scores for donor and acceptor sites allows comparisons with other tools, such as SpliceAI, which assess splice sites individually.*

*Following is the new paragraph added in the Discussion section:*

*“In the biological context, donor and acceptor sites are not independent. Therefore, rather than training separately on the two types of splice sites, Splam is designed and trained to recognize pairs of donor and acceptor sites, mimicking the behavior of the spliceosome. Although there is a strong correlation between Splam's donor and acceptor site scores, the model is intentionally designed to produce distinct scores for each. This design can be useful in cases where the scores differ, suggesting that one of the sites might be weaker than the other.”*

***=== Response 2.2 end ===***

The paper also addresses another important shortcoming of SpliceAI which is that output values tend to cluster around zero, even for true splice sites, so the approach is highly sensitive to the choice of threshold. In contrast, the output values in Splam cluster more heavily around 1 and zero, making them a lot less sensitive on the choice of threshold.

The paper also shows that Splam generalizes much better to different species, including species as far removed from humans as Arabidopsis thaliana.

I feel less qualified to evaluate the proposed application of filtering spliced alignments on transcriptome assembly.

*Thanks for the positive feedback!*

**2.3** For me, one of the biggest questions I had in evaluating this work was the choice of negative splice junctions. The authors make a strong attempt to avoid including pairs that may actually be spliced, but this risks excluding from the negative datasets more ambiguous pairs. I was especially concerned by Figure 6b, because it seems to suggest that the positive and negative sites look quite different for both acceptors and donors. Is this a misreading of Figure 6?

One problem is in the strong distinction that this model makes between positive and negative pairs, when in practice there are a lot of pairs with varying levels of evidence for whether or not they bound a possible intron. One experiment I would really like to see is one where you take all possible pairs of acceptor and donor sites within a set of genes, and then you assign a score based on the evidence you have that this pair represents an intron. So all the pairs that are part of your negative dataset would be close to zero, but many other pairs would have scores in between zero and one. Then I would like to see a plot that has this score in the x-axis and the output produced by your model in the y-axis.

I would really like to see more evidence that the strong results in the paper are not an artifact of the conservative choice of negative datasets. The worry is that there are many acceptor donor pairs that in practice have very little chance of marking an intron but are excluded from the negative sets and therefore make the false positive rate seem lower than it really is.

***=== Response 2.3 begin ===***

*In response to this concern, we have run new sets of experiments evaluating Splam's performance as a function of the amount of evidence, measured by the number of spliced reads supporting each putative splice junciton. We have described these experiments in a new Supplementary note,* ***Note 2****. We also created 2 new figures (****Figure S11, S12****), added paragraphs to the Methods and Results 2.3 section, and updated* ***Figure 6****. The note and figures were included into the supplementary file. All of these changes are described below.*

(1) ***Following is Note S2 which we have added to the supplementary file:***

***Note S2.*** *Evaluation of Splam on unannotated splice junctions extracted from GTEx RNA-Seq samples with varying levels of spliced alignment support.*

*We collected unannotated splice junctions from 9,795 GTEx RNA-Seq experiments used to create the CHESS human database ^11^, supported by varying numbers of spliced alignments, ranging from 2 to 100 reads. We chose junctions from chromosome 1 and chromosome 9, which were excluded from the training dataset. The datasets are named Novel-2, Novel-3, etc. up to Novel-100, indicating the number of supporting spliced alignments. This selection process was intended to assess Splam's performance across a wide array of negative splice junctions, from those highly unlikely to be true splice sites (with just 2-3 reads supporting them) to those with much greater support.*

*We applied Splam to score each dataset, from Novel-2, Novel-3, and all subsequent datasets through Novel-100. The donor and acceptor score distributions for selected datasets (Novel-2, Novel-20, Novel-40, Novel-60, Novel-80, and Novel-100) are presented in Figure S11. As the number of spliced alignments supporting the splice junctions increases, there is a more notable peak at the score of 1.0, indicating that Splam is more likely to identify these sites as genuine splice donors or acceptors.*

*Next, to compare splice sites with high Splam scores to those with low scores, we set a threshold of 0.5 and quantified the proportion of splice junctions that Splam predicted as positive. Figure S12a shows that Splam predicts a greater number of splice junctions as positive when they are supported by a higher number of spliced alignments. We also calculated the average donor and acceptor site scores for each Novel-x group and plotted them against the number of spliced alignments (Figure S12b). The mean splice site scores are also proportional to the number of spliced alignments. These result show that as the number of spliced alignments increases, Splam is more likely to assign a high score to the junction, despite Splam being trained solely on DNA sequences without any coverage information.*

(2) ***Following are Figure S11 and S12 added in the supplementary file:***

**Figure S11:** Frequency distribution and kernel density plots of donor and acceptor site scores. The left column presents the donor site score distribution, and the right column shows the acceptor site score distribution for: (a) Novel-2, (b) Novel-20, (c) Novel-40, (d) Novel-60, (e) Novel-80, and (f) Novel-100.

**Figure S12:** Results of evaluating unannotated splice junctions across GTEx-supported datasets with varying spliced alignment counts. (a) Scatter plots depict the ratio of splice sites with Splam scores above 0.5 for donor (left) and acceptor (right) sites relative to their respective spliced alignment counts. (b) Scatter plots display the mean Splam scores for both donor (left) and acceptor (right) sites compared to their respective spliced alignment counts. In each plot, the trend line is illustrated in red.

(3) ***Following are the updated Figure 6 and new paragraphs added in Methods and Results sections:***

***In Methods section:***

**Figure 6: (a)** Illustration of the four types of splice sites used for training and testing Splam: Positive-MANE, Positive-Alt, Negative-1, and Negative-Random. Positive-MANE sites (blue) are selected from the MANE database and supported by at least 100 alignments, while Positive-Alt sites (green) are present in the RefSeq database but missing from MANE, and also supported by at least 100 alignments. Negative-1 sites (orange) occur on the opposite strand of a known gene and are supported by only 1 alignment, and Negative Random sites (red) are random GT-AG pairs on the opposite strand that do not overlap with any known splice sites and have no alignment support. **(b)** DNA logos for donor and acceptor site for Positive-MANE, Positive-Alt, Negative-Random, Negative-1, Novel-50, and Novel-100 display the conservation of residues at each position in the training set created for this study. Each panel displays 40 nucleotides, centering on the donor and acceptor sites. The y-axis represents the level of conservation, measured in bits, with 0 representing equal probability among “A”, “C”, “G”, and “T”, and 2 representing a single nucleotide dominating the position.

***“5.1 Generating high-quality datasets for splice junctions***

*We curated two sets of positive examples, “Positive-MANE” and “Positive-Alt”, and two sets of negative examples, “Negative-1” and “Negative-Random”, to serve as high-quality datasets for training and testing the Splam model. Additionally, we prepared a series of testing datasets, from Novel-2 through Novel-100, to evaluate splice junctions with varying levels of spliced alignment support. DNA logos for these datasets are presented in Figure 6a.*

*5.1.3 RNA-Seq novel junction dataset creation*

*In practice, RNA-Seq alignments contain many unannotated splice junctions with different levels of evidence suggesting they may bound a possible intron. To evaluate Splam's recognition of "negative" splice junctions, we expanded our test dataset by adopting the same criteria used for the Negative-1 but varying the levels of spliced alignment support. This led to datasets named Novel-2 through Novel-100, based on the number of supporting spliced alignments. It is important to note that these datasets were solely used for evaluating Splam and were not seen during the training process. The underlying logic for this approach is that splice junctions with a greater count of biological read supports are more likely to represent unannotated novel splice junctions.*

***5.1.4 Evaluating the donor and acceptor sites in positive and negative datasets***

*Figure 6b shows DNA logos for each of our positive and negative datasets, showing the sequence composition and conservation levels. The donor and acceptor patterns for Positive-MANE and Positive-Alt are almost identical, indicating that splice junctions in MANE and alternatively spliced transcripts from RefSeq have the same consensus, as expected for positive examples. The two negative datasets show almost no pattern of conservation, although the Negative-1 group has a very small amount of conservation immediately adjacent to each splice junction. The two groups of negatively labeled splice junctions are clearly distinguishable from the annotated true splice sites. For the Novel-x datasets, DNA logos from Novel-50 and Novel-100 categories show slight differences compared to Negative-1, especially in the acceptor sequences, where faint signals of a polypyrimidine tract appear upstream of the conserved AG acceptor motif. This indicates that while these unannotated junction sets are mostly negative, a small portion of positive splice junctions might exist within the Novel-50 and Novel-100 datasets. Table S12 reports the numbers of dinucleotides at the donor and acceptor sites, and Figure S15 illustrates the ratio of canonical to non-canonical splice sites.”*

***In Results section:***

***2.3 Effect of filtering low-scoring spliced alignments on transcriptome assembly***

*“One crucial step in many transcriptome analyses involves aligning spliced reads to the genome, using an alignment program such as HISAT2 ^12^ or STAR ^13^. The output from these programs might contain two types of erroneous alignments: (1) incorrect alignments due to sequencing errors, repeats, or other sequence artifacts (computational noise), and (2) correct alignments of reads that result from noisy splicing (biological noise). Either computational or biological noise ^14^ can lead to systematic underestimates of transcript abundance levels and large increases in the number of false positive genes and transcripts ^15^. We reasoned that Splam could distinguish novel splice junctions from both types of noisy junctions, which would receive low scores from Splam; thus, to assess Splam's efficacy on ambiguous, unannotated splice junctions in RNA-Seq alignments, we created a series of unannotated splice junction datasets with varying levels of support, ranging from 2 to 100 alignments, as described in the Methods section. Our observations showed that while those splice junctions were predominantly predicted as negative, junctions supported by a higher number of spliced alignments receive higher average scores from Splam (see Supplementary Note S2 and Figure S11 and S12).*

*We also evaluated the effectiveness of using Splam to improve the quality of transcriptome assemblies by removing low-scoring splice junctions. Using the workflow illustrated in Figure 5 and further detailed in Methods, we processed 20 randomly selected RNA-seq samples, of which 10 were prepared using a poly-A capture library preparation, and 10 were prepared using ribosomal RNA depletion.”*

***=== Response 2.3 end ===***

Smaller issues.

**2.4** The manuscript seems to be missing a supplement. There are many different references to figures S1, S2, but I did not see them.

***=== Response 2.4 begin ===***

*We've confirmed that a supplementary file was submitted with the main manuscript but may have been lost due to a system error.*

*We have updated the supplementary file in the revised submission.*

***=== Response 2.4 end ===***

**2.5** There is also a reference to a "Figure 3c-d" but there is no figure 3 (d) in the paper.

***=== Response 2.5 begin ===***

*We have revised the manuscript text to accurately reflect this merged figure.*

*Following is the corrected paragraph from the revised manuscript:*

*“Our findings indicate that Splam consistently captures more splice junctions compared to SpliceAI across all thresholds. Furthermore, Splam outperforms SpliceAI in distinguishing splice junctions in* *alternatively spliced isoforms (Figure 3c). Not surprisingly, Splam exhibits even larger improvements when compared to SpliceAI-10k-Ns, as illustrated in Figure S4, Figure S5, Figure S6, and Figure S7.”*

***=== Response 2.5 end ===***

***Reference***

*1.	Jaganathan, K. et al. Predicting splicing from primary sequence with deep learning. Cell* ***176****, 535-548. e524 (2019).*

*2.	Zeng, T. & Li, Y.I. Predicting RNA splicing from DNA sequence using Pangolin. Genome biology* ***23****, 103 (2022).*

*3.	Scalzitti, N. et al. Spliceator: multi-species splice site prediction using convolutional neural networks. BMC bioinformatics* ***22****, 1-26 (2021).*

*4.	Chen, K. et al. Self-supervised learning on millions of pre-mRNA sequences improves sequence-based RNA splicing prediction. bioRxiv, 2023.2001. 2031.526427 (2023).*

*5.	Akpokiro, V., Martin, T. & Oluwadare, O. EnsembleSplice: ensemble deep learning model for splice site prediction. BMC bioinformatics* ***23****, 413 (2022).*

*6.	Albaradei, S. et al. Splice2Deep: An ensemble of deep convolutional neural networks for improved splice site prediction in genomic DNA. Gene* ***763****, 100035 (2020).*

*7.	Scalzitti, N. et al. Spliceator: Multi-species splice site prediction using convolutional neural networks. BMC bioinformatics* ***22****, 1-26 (2021).*

*8.	Kelley, D.R. Cross-species regulatory sequence activity prediction. PLoS computational biology* ***16****, e1008050 (2020).*

*9.	Wang, R., Wang, Z., Wang, J. & Li, S. SpliceFinder: ab initio prediction of splice sites using convolutional neural network. BMC bioinformatics* ***20****, 1-13 (2019).*

*10.	Avsec, Ž. et al. Effective gene expression prediction from sequence by integrating long-range interactions. Nature methods* ***18****, 1196-1203 (2021).*

*11.	Varabyou, A. et al. CHESS 3: an improved, comprehensive catalog of human genes and transcripts based on large-scale expression data, phylogenetic analysis, and protein structure. Genome Biology* ***24****, 1-16 (2023).*

*12.	Kim, D., Paggi, J.M., Park, C., Bennett, C. & Salzberg, S.L. Graph-based genome alignment and genotyping with HISAT2 and HISAT-genotype. Nature biotechnology* ***37****, 907-915 (2019).*

*13.	Dobin, A. et al. STAR: ultrafast universal RNA-seq aligner. Bioinformatics* ***29****, 15-21 (2013).*

*14.	Varabyou, A. et al. CHESS 3: an improved, comprehensive catalog of human genes and transcripts based on large-scale expression data, phylogenetic analysis, and protein structure. bioRxiv, 2022.2012. 2021.521274 (2022).*

*15.	Varabyou, A., Salzberg, S.L. & Pertea, M. Effects of transcriptional noise on estimates of gene and transcript expression in RNA sequencing experiments. Genome research* ***31****, 301-308 (2021).*

**Second round of review**

**Reviewer 1**

Satisfactory. The authors have addressed all my previous comments.

**Reviewer 2**

The authors did a good job of addressing the major concern that I had with the manuscript, and their new edits improve the paper significantly. More detailed comments and a few pending questions are included below.

First, I appreciate the new data. For me, the new experiments address the most important concern I had, which was that the negative set was "too easy" for the model, so that the model would rate as positive anything that looked like a plausible junction. The results on the Novel_x datasets show that this is not the case, and indeed, they show that even with high values of x, the model produces mostly zero scores.

However, the new experiment also raised another question for me. If I understand correctly the methodology for how the different datasets were built, the Novel_100 dataset, which would contain junctions supported by 100 reads from GTEx, sounds a lot like how the Positive_MANE dataset was constructed, so I was very surprised to see the model output zero on almost the entire Novel_100 dataset. I think it is important to say something about why this is. Is this simply that for Positive_MANE, 100 is a lower bound on the number of GTEx reads, so that most of Positive_MANE has many more than that? If that's the case, it would be useful to expand the range of this experiment, to bigger values of x so one can observe the transition from when the model is outputting mostly zero, to when the model is outputting mostly one.

In describing the graphs in S12, the paper makes a big deal about the fact that there is some correlation between the ratio of splice junctions with Splam scores above threshold and the Spliced alignment count, but I think it's important to point out in the paper that even at 100, the ratio barely gets to 0.12. Following up on the earlier comment, I think this experiment would be even more helpful if the range was extended beyond 100.

But overall, as I said before, this experiment addressed the main concern I originally had about the paper.

I also have some questions about the figures in the supplement which I didn't get to see in the first round; especially Figures S2a, raises some questions for me.

For the SPLAM input/output figure, I was surprised about the output. The figure seems to suggest that the output in fact has an entry for each of the 400 entries in the input. Why? Do you do anything

with the predictions for the nucleotides not at the center of the window? From everything I understood from the paper, I suspect this is just a bad picture, and that you actually only have one output (a 3d vector) at the donor and acceptor sites, but if that's not the case, then this needs to be explained better.

And then for the SpliceAI input/output figure, that looks really different from what I expected to see. SpliceAI takes a 10k+1 window around the candidate splice point and produces a 3d vector for that site, so why are you computing outputs for every site in the entire intron? This is also relevant to figure S2b, if you are counting as part of the input to SpliceAI the entire intron length, that seems unnecessary, except of course for when the intron is less than 5000nt long. It would be good to clarify this question about inputs and outputs.

I think these are just presentation issues, but they should be clarified.

Finally, with regards to my question in the original review about the quadratic nature of the algorithm. I was happy with the response, but I think it is important to emphasize more in the paper that this system is solving a different problem from what SpliceAI was originally intended to solve. SpliceAI was intended for exhaustive scanning of entire genes to identify likely canonical splice sites. It's a different problem, as the authors point out in the new text. This needs to be made clearer early on to higlight that while SpliceAI is it's main baseline, this paper is solving a different problem from what SpliceAI was designed to solve.

I would like these points to be addressed in a final paper, but overall I am happy with the changes and I think this is a valuable contribution to the space of AI for splicing.

**Authors Response**

**Point-by-point responses to the reviewers’ comments:**

**Referee 2:**

1.	First, I appreciate the new data. For me, the new experiments address the most important concern I had, which was that the negative set was "too easy" for the model, so that the model would rate as positive anything that looked like a plausible junction. The results on the Novel_x datasets show that this is not the case, and indeed, they show that even with high values of x, the model produces mostly zero scores.

 However, the new experiment also raised another question for me. If I understand correctly the methodology for how the different datasets were built, the Novel_100 dataset, which would contain junctions supported by 100 reads from GTEx, sounds a lot like how the Positive_MANE dataset was constructed, so I was very surprised to see the model output zero on almost the entire Novel_100 dataset. I think it is important to say something about why this is. Is this simply that for Positive_MANE, 100 is a lower bound on the number of GTEx reads, so that most of Positive_MANE has many more than that? If that's the case, it would be useful to expand the range of this experiment, to bigger values of x so one can observe the transition from when the model is outputting mostly zero, to when the model is outputting mostly one.

In describing the graphs in S12, the paper makes a big deal about the fact that there is some correlation between the ratio of splice junctions with Splam scores above threshold and the Spliced alignment count, but I think it's important to point out in the paper that even at 100, the ratio barely gets to 0.12. Following up on the earlier comment, I think this experiment would be even more helpful if the range was extended beyond 100.

 But overall, as I said before, this experiment addressed the main concern I originally had about the paper.

*Response: The vast majority of splice junctions seen in the GTEx dataset are predominantly supported by just one or very few spliced alignments (i.e., they are noise or alignment artifacts). For example, out of 100,000 randomly selected spliced reads, 65,568 have coverage of one, and 12,081 have coverage of two.*

*In contrast, splice junctions in the MANE dataset have extremely deep coverage when we sum it up across all of GTEx.* ***The mean number of spliced alignments supporting splice junctions is 871,292, and the median is 235,110****. Thus, a support level of 100 spliced reads is at the extreme lower end of the distribution for the MANE annotation.*

*To extend the experiment, we extracted splice junctions supported by >10,000 spliced alignments in GTEx dataset and excluded those present in the MANE annotation. In total, there are 149,901 such junctions. We scored these using Splam, finding that the mean donor and acceptor site scores are 0.66, with medians of 0.99. Among these deeply-covered splice junctions, 66.7% scored above 0.5 for both sites. Thus the trend we reported does continue at much higher levels of coverage.*

*To briefly summarize this in the manuscript, we edited the result section as follows:*

*To assess Splam's efficacy on ambiguous, unannotated splice junctions in RNA-seq alignments, we created a series of unannotated splice junction datasets with varying levels of support, ranging from Novel-2 to Novel-100, as described in the Methods section. Our observations showed that while splice junctions are predominantly predicted as negative junctions for those with low spliced alignment support, those supported by a higher number of spliced alignments receive higher average scores from Splam (see Supplementary Additional file 1: Note S2 and Additional file 1: Fig S11 and S12). We also evaluated the splice junctions supported by over 10,000 spliced alignments in the GTEx dataset, excluding those present in the MANE annotation, which comprised 149,901 junctions. Splam tended to give these well-supported junctions relatively high scores, with a mean score of 0.66 for both donor and acceptor sites, and 66.7% of these junctions scored above 0.5 for both sites.*

2.	I also have some questions about the figures in the supplement which I didn't get to see in the first round; especially Figures S2a, raises some questions for me.

 For the SPLAM input/output figure, I was surprised about the output. The figure seems to suggest that the output in fact has an entry for each of the 400 entries in the input. Why? Do you do anything with the predictions for the nucleotides not at the center of the window? From everything I understood from the paper, I suspect this is just a bad picture, and that you actually only have one output (a 3d vector) at the donor and acceptor sites, but if that's not the case, then this needs to be explained better.

 And then for the SpliceAI input/output figure, that looks really different from what I expected to see. SpliceAI takes a 10k+1 window around the candidate splice point and produces a 3d vector for that site, so why are you computing outputs for every site in the entire intron? This is also relevant to figure S2b, if you are counting as part of the input to SpliceAI the entire intron length, that seems unnecessary, except of course for when the intron is less than 5000nt long. It would be good to clarify this question about inputs and outputs.

I think these are just presentation issues, but they should be clarified.

*Response:* *First, we should clarify that both Splam and SpliceAI produce outputs for every single base in the input window, as shown in Figure S2a. This is simply a part of the deep neural net architecture used by both programs. Even though both programs produce hundreds (Splam) or thousands (SpliceAI) of outputs, though, our evaluation only looked at the scores for a single donor-acceptor pair for each example.*

*Splam's model scores each position within the 800bp input window, and our evaluation program only looks at the 200^th^ and 600^th^ positions as donor and acceptor site scores. These two scores determine if a splice junction is valid.*

*Since we are evaluating at the splice junction level, we provided SpliceAI with a super-set of the splice junction information that Splam used. SpliceAI by design requires a continuous sequence as input, and it outputs scores for all positions. Therefore we had to give it the entire intron plus another 200bp beyond each splice site (the same as what Splam saw), plus another 5000bp on each side flanking the splice sites. The extra flanking sequence is required by SpliceAI's algorithm. It then outputs scores for all positions. For evaluation, we only extracted the scores for the donor and acceptor sites.*

*To clarify this question, we have edited the manuscript as follows (updates highlighted:*

*(in the section* ***Splam model architecture design****)*

*The model design of Splam mainly follows the architecture of SpliceAI with some optimizations. We utilized a deep residual convolutional neural network (CNN) that incorporates grouped dilated convolution layers within the residual units (Figure 1a). The input to the model is 800nt of one-hot encoded DNA sequence, with the dimension of 3 × 800, and the output is a 3 × 800 array with the predicted probability that each base is a donor site, acceptor site, or neither (Methods 5.2). The Splam program outputs the nucleotide scores at positions 200 and 600 as the donor and acceptor scores, respectively. We constructed the Splam model using PyTorch framework version 1.13.0[53].*

*(in the section* ***Formatting SpliceAI input and output data****)*

*The SpliceAI model requires a 10,000nt context to run, meaning that the input sequence needs to be padded with 5,000nt of flanking sequence on either side. We included the full intron sequence plus an additional 200nt before and after, to ensure that SpliceAI had at least as much input sequence as Splam. In the “SpliceAI” version, we extracted the full 5.2Kb flanking sequence from the genome (Additional file 1: Fig S2a). In the “SpliceAI-10k-Ns” version, we replaced the outer 5,000nt on each flanking sequence with Ns (zero-padding, as outlined in[23]), leaving only a 200nt sequence from the genome. Although SpliceAI outputs scores for every position inside the 10Kb flanking sequences, for evaluation we only extracted the scores for the donor and acceptor sites.*

**3.**     Finally, with regards to my question in the original review about the quadratic nature of the algorithm. I was happy with the response, but I think it is important to emphasize more in the paper that this system is solving a different problem from what SpliceAI was originally intended to solve. SpliceAI was intended for exhaustive scanning of entire genes to identify likely canonical splice sites. It's a different problem, as the authors point out in the new text. This needs to be made clearer early on to higlight that while SpliceAI is it's main baseline, this paper is solving a different problem from what SpliceAI was designed to solve.
*Response: We have revised the introduction to clarify this point. Our revised text appears at the end of the Background section, and reads as follows:*

*In this paper, we introduce Splam, a new deep learning approach to splice site prediction in which the system learns to recognize paired donor and acceptor sites from genomic sequences. In comparison to SpliceAI, our method uses a much shorter flanking context sequence to predict splice sites, which is more biologically realistic. Unlike SpliceAI, which was designed to predict a single canonical set of splice sites for each gene, Splam is able to predict the splice sites from multiple distinct isoforms for a single gene locus. In our experiments, we demonstrate that Splam is more accurate than SpliceAI, and we also show how it can be used to improve the accuracy of genome-guided transcript assemblers by removing spurious alignments produced by spliced aligners.*
